# Case of Apoplexy of the Spleen

**Published:** 1830-07-01

**Authors:** 


### XXXV.

### CASE OF APOPLEXY OF THE SPLEEN. By Mr. HENRY ANCILL.

George Golding; a labouring blacksmith, 39 years of age; an habitual drunkard, his favourite liquor being rum, of which he would ordinarily take from half a pint to a pint, and occasionally a pint and a quarter before breakfast; his appetite was in general uncommonly good;—bowels regularly evacuated every morning about six o'clock, and he is said to have been a hard working man, of a mild temper, and inoffensive habits.

He suffered under an attack of hemiplegia ten or eleven years ago at Whitsuntide holidays, which was removed after three weeks medical attendance, the principal remedies employed, having been, a very copious bleeding, purging, friction and warmth; the only remnant of the affection was a slight distortion of the mouth sideways.

Two months ago he had an illness which is stated to have been pleuritis, seated in the right side, and was subdued in fourteen days, by repeated bleeding and the usual remedies:—since this his health gradually improved, he got fat, and latterly his appetite was so great as to become a subject of remark with his fellow workmen.

*On Friday morning, April 30th;* he rose at the usual hour perfectly well, the bowels acting soon after; he then went to his ordinary labour and continued at it until the moment of the present attack, which occurred on leaving off, for the purpose of taking his breakfast; in the course of the morning he had remarked that he never felt better in his life, and drank in two portions a quarter of a pint of rum in a pint of porter.

*At half past 9.* I was called to visit him; I saw him first sitting in a bent position upon a low seat close to the forge, his head resting on his arms, and his arms upon his knees; the statement made, was that he had been suddenly seized a few minutes before with the symptoms he then laboured under; these were, nausea, a sense of weight in the epigastrium, most profuse perspiration, pale face, extremities below their natural temperature, and an undulating pulse of about 120 in a minute, full, soft and regular; in reply to enquiries I was informed that his first complaint was of nausea, but he did not vomit, and that the appearance of the face was natural; he was immediately conveyed to his lodgings.

*Ten o'clock* A.M.; examined in a recumbent position. The perspiration has now subsided; great pain is felt upon pressure, in the epigastrium extending to the umbilicus and throughout the left hypochondriac region; the abdomen is rather warmer than natural; pulse 130, stronger and still perfectly regular; the other symptoms remain the same.

Sumat. statim hydrarg. submur. gr. vj.

℞ Sodæ sulphat. ℥i.

Infusi sennæ ℥ iv.

Decoct. farinæ ex semin. avenæ ℥viij.

ut fiat enema statim adhibendum.

His wife was directed to send a statement of his feelings after the return of the injection which she failed to do.

*Six o'clock* P. M. The bowels have acted six times from the operation of the purgatives, and there has been a free discharge of urine, the appearance of both are natural; he now complains of violent pain diffused over the whole abdomen, but most particularly seated in the above described regions, and greatly aggravated by pressure; is most easy in a bent position, or as he terms it, when *doubled up,* and is still capable of exertion, moving on and off the bed without assistance: there is now also an appearance of fulness in the left hypochondriac and iliac regions; great thirst; tongue very dry; no perspiration; the respiration somewhat laborious; the trunk of the body is warmer, but the extremities below their natural temperature, and the pulse 140—in other respects as at 10 o'clock.

These symptoms approaching nearer in character to peritonitis than any other disease—a free opening was made into the basilic vein, the blood at first flowed freely, but gradually, after bleeding a few ounces; and in the space of about two minutes ceased.

Upon reviewing the case it was now referred to hemorrhage into the abdominal cavity and all hopes of relief given over.

*Nine o'clock* P.M. Complains of excessive pain over the whole abdomen, the respiration is more laborious; extremities cold; lips and countenance exsanguine, and the pulse sinking.

℞ Tincturæ opii ♏ xxxv.

Spiritus æther. sulph.

lavendulæ c. āā ʒss.

Misturæ camphoræ ℥xss. misce ut ft. haustus statim sumendus.

*May 1st, 1 o'clock,* A. M. Mortuus est; being about 16 hours from the commencement of the attack.

*Post mortem examination by Mr. Alexander Thomson.* The *body* was remarkably well proportioned, plump and solid. *Thorax,* resounded well in every part upon percussion. *Left leg,* had the remains of an impetiginous eruption upon its anterior and exterior side. HEAD.—The *integuments* adhered firmly to the pericranium; the *cranium* was thick and ivory-like in its texture, having very little diplöe; *dura mater* had its minute arteries considerably more injected than usual, the *arachnoid membrane,* was separated from the pia mater by a considerable quantity of colourless serum and was opaque in every direction; a great deal of colourless serum was also seen between the convolutions of the cerebrum and cerebellum; *the pia mater* adhered so laxly to the substance of the brain as to be removable by the slightest effort, its arteries were full but its veins destitute of blood; the *cerebrum* was unusually firm and unyielding both in its cineritious and medullary portions, and presented but few red points in its sections; the *nerves* were all remarkably firm and white at their origins; the *cerebellum* was very soft and apparently destitute of elasticity, it yielded easily to the pressure of the fingers; the *pons varolii* and *medulla oblongata* were nevertheless exceedingly hard; the *lateral ventricles* were greatly dilated and filled with a limpid almost colourless serum, yet the processes of cerebral matter in them were unusually firm; the *veins* of the fornix contained blood, but those of the plexus choroides, and the vena magna galeni were quite empty, as were also the arteries of the plexus; the *pineal gland* was larger than usual, it contained in its capsule some limpid fluid and one hard, round, smooth, strong, concretion; the *third* and *fourth ventricles* also contained fluid of a description similar to that in the lateral ventricles; and there was a considerable quantity in the *base* of the *cranium.*

THORAX. The *muscles* of the trunk were very pale, and together with the *cellular tissue* remarkably destitute of blood; *the lungs,* though covered with rather more sub-pleural black spots and reticulations than usual, were yet very healthy; they adhered slightly by old adhesions to the upper part of the parietes of the thorax on both sides and to the diaphragm, having each, at about the centre of the lateral aspect, a small puckering of pleural investment resembling a cicatrix, and within this a few condensed and indurated lobules, as though there had been small cavities which were obliterated; the *pericardium* was healthy though unusually loaded on the exterior with fat, and it contained about six drachms of greenish transparent serum: the *heart* was natural in size, greatly loaded along the course of its vessels with fat, its substance of an exceedingly pale yellowish white colour, flabby, lacerable and adhesive to the fingers; the *lining membrane* of all its cavities was opaque in many parts; the *mitral* and tricuspid valves, were opaque but not thickened, though the ligaments surrounding the auriculo-ventricular orifices were indurated and semicartilaginous in some spots; the *semilunar valves* both of the pulmonary artery and of the aorta were opaque: those of the former not being thickened: those of the latter thickened and semicartilaginous: all were imperfect round their attachments having greater or less perforations towards their free and floating margins:—that these perforations were not recent was evident, because their edges were not ragged, because they could not be extended by considerable force, and because their polished smooth surface was traced to pass continuously over the edges of these orifices.

ABDOMEN. When cut into it discharged a great quantity of dark-coloured bloody serum, and when opened it displayed an enormous black coagulum investing and attached to the lower or floating margin of the great omentum, extending from the right hypochondriac, through the umbilical and into the left hypochondriac regions. In the last situation it was materially increased in size, so that when carefully separated it would have filled a pint pot: upon the whole of this extraordinary mass being removed, a small laceration not larger than the tip of the finger, through the peritoneum and tunic of the spleen was observed at a spot where this organ had become attached by its superior edge to the diaphragm:—no other aperture could be ascertained in the peritoneum; the *spleen*, which was now carefully examined appeared enormously large, it was attached superiorly to the diaphragm, by the whole of its concave surface to the cul-de-sac of the stomach, and by the greater part of its external superficies also to the diaphragm. When carefully removed and cut across, its substance appeared lacerated into large angular fragments, which were kept together by a cement of dark black coagulum;—the natural inference from which was, that the vessels of the substance of this organ had given way, an opinion strengthened by the fact, that this cementing portion of coagulum was continuous with a mass an inch and a half thick of the same substance which invested the whole of the organ, between the parenchyma of which and its fibrous envelope, it had been deposited: the substance of the angular fragments yielded to the slightest pressure of the fingers, and broke down into a soft pap-like mass, so that the vessels could not be traced:—the spleen in truth was not enlarged, but its vessels having given way, the blood was discharged into its substance,—tore it into fragments,—separated its parenchyma from its fibrous envelope, which it first violently distended and then ruptured in two places, at its upper and at its most depending part; for from the latter the blood had escaped in large quantity, and separated the two layers of the descending mesocolon from each other, to the distance of an inch and a half, as far down as the sigmoid flexure; the *liver*, was remarkably large, externally firm, and of a mottled appearance like a piece of granite, internally of a dirty pale buff-yellow hue, and easily broken into granules by pressure; the *gall bladder* was distended with bile of a natural colour; the *mesenteric and intestinal vessels*, were more than ordinarily destitute of blood; the *intestines* healthy; the *stomach* contained, apparently unchanged, some gruel which he had taken shortly before his death, all its coats were remarkably thin, and towards the cardiac orifice its villous coat had several patches about the size of half-a-crown, covered densely with stellæ of red vessels; the *pancreas* and all the rest of the viscera presented their usual characteristics.

*Oxford Street, May*, 1830.

N.B. The above dissection, so ably and minutely described, is interesting in many points of view, as shewing the state of organs in a man habituated to so much strong drink, and cut off apparently in the midst of health.—*Ed.*

